# Effect of Pullulan Coating on Postharvest Quality and Shelf-Life of Highbush Blueberry (*Vaccinium corymbosum* L.)

**DOI:** 10.3390/ma10080965

**Published:** 2017-08-18

**Authors:** Karolina Kraśniewska, Iwona Ścibisz, Małgorzata Gniewosz, Marta Mitek, Katarzyna Pobiega, Andrzej Cendrowski

**Affiliations:** 1 Division of Biotechnology and Microbiology, Department of Biotechnology, Microbiology and Food Evaluation, Warsaw University of Life Sciences-SGGW, Nowoursynowska 159c, 02-776 Warsaw, Poland; malgorzata_gniewosz@sggw.pl (M.G.); katarzyna_pobiega@sggw.pl (K.P.); 2 Division of Fruit and Vegetable Technology, Department of Food Technology, Warsaw University of Life Sciences-SGGW, Nowoursynowska 159c, 02-776 Warsaw, Poland; iwona_scibisz@sggw.pl (I.Ś.); marta_mitek@sggw.pl (M.M.); andrzej_cendrowski@sggw.pl (A.C.)

**Keywords:** pullulan, coating, blueberry fruit, *Vaccinium corymbosum* L., shelf-life, postharvest quality

## Abstract

Fruits form an important part of a healthy human diet as they contain many ingredients with proven pro-health effects such as vitamins, phenolic compounds, organic acids, fiber, and minerals. The purpose of this work was to evaluate the effect of pullulan coating on the quality and shelf life of highbush blueberry during storage. General appearance, weight loss, dry matter, soluble solid content, reducing sugars, content of L-ascorbic acid, phenolic compounds (total phenolics, phenolics acids and anthocyanins) were determined in uncoated and coated blueberries fruits. The microbiological efficiency of pullulan coating was also evaluated. All parameters were monitored during storage at 4 °C and 16 °C by 28 and 14 days, respectively. The study showed that pullulan coating protects perishable food products especially susceptible to mechanical injury including fruits such as blueberries. Pullulan acts as a barrier that minimizes respiration rate, delaying deterioration and controlling microbial growth.

## 1. Introduction

Fruits form an important part of a healthy human diet as they contain many ingredients with proven pro-health effects such as vitamins, phenolic compounds, organic acids, fiber, and minerals. Natural antioxidants—such as vitamin C, anthocyanins, and polyphenols—which play an important role in the prevention of development of civilization diseases, are particularly important in human nutrition [1,2,3]. Berries are fruits with high antioxidant activity and, among them, the highbush blueberry (*Vaccinium corymbosum* L.) is highly desirable [4,5,6]. Poland is the fourth (after the United States, Canada, and France) largest producer of the highbush blueberry [7]. Popularity of this fruit among consumers is constantly increasing due to its high nutritional value and good sensory qualities. These fruits are rich in polyphenols that are antioxidants, especially anthocyanins, flavonols, catechins, and phenolic acids [6]. Introduction of blueberries into our diet shows positive health benefits such as prevention of cardiovascular diseases, cancer, arthritis, and autoimmune diseases [8,9,10].

In general, fresh fruits are characterized with increased metabolic activity which reduces their shelf life. The majority of the qualitative and quantitative losses of fruits occur between the times of their harvest and consumption. An important condition for maintaining the stability of such fruits is proper storage and control of their quality parameters (water content, weight loss, texture change, color, and firmness). The primary causes of fruit spoilage and reduced stability are respiration and transpiration, as well as the growth of microflora, in particular fungal and bacterial species [11,12]. Therefore, new technologies are being developed that delay the postharvest deterioration of fruits resulting from biochemical, physical, and microbial activity and those that improve their stability and storage. One such technique to preserve the quality of fruits is the use of thin layers of edible coatings on the surface of the fruit. These coatings are intended to control the exchange of gases between the fruit and the environment. This helps to reduce weight loss and to preserve the composition of the internal atmosphere of the fruit, thereby slowing down metabolic processes and prolonging its shelf life [12,13,14]. 

As so far, there are a few works about coating effects on blueberry fruit. According to this research, edible coatings such as chitosan, starch, pectin, sodium alginate, and calcium caseinate have exhibited beneficial roles in maintaining quality of berries. Coating has been successfully used to controlling the decay and extending the shelf life of postharvest blueberries. Furthermore, the use of coating had positive effects on weight loss, firmness, titratable acidity; maintained surface lightness; and delayed the decrease in anthocyanin content, phenolic content, and antioxidant capacity of coated fruit products [13,15,16,17].

Currently, research is underway on the use of pullulan coatings to increase the shelf life of fruits. Pullulan coating has good adhesive properties, high mechanical strength, and does not react with food ingredients. In addition, pullulan coatings are colorless, tasteless, and odorless and have limited permeability to gases such as oxygen and carbon dioxide [18,19,20,21]. In addition, pullulan is a hard-to-absorb carbon source that is not used by bacteria and fungi that are responsible for spoilage of food [22]. Pullulan coatings have been used for, extending the shelf life of apples (whole and in pieces), strawberries, kiwifruit, carrots, peppers, and Brussels sprouts [23,24,25,26,27].

The purpose of this work was to evaluate the effect of pullulan coating on the quality and shelf life of highbush blueberry during storage. We evaluated the influence of pullulan coating on qualitative and quantitative losses of blueberries stored at refrigerator temperature (4 °C) and commercial temperature (16 °C) for 28 and 14 days, respectively. The changes in weight loss, dry matter, soluble solid content, reducing sugars, and microbiological stability of the fruit were studied. Due to the health benefits of blueberry fruit, the influence of pullulan coating on changes in L-ascorbic acid and phenolic compounds (total phenolics, phenolics acids and anthocyanins) was also evaluated.

## 2. Results and Discussion

### 2.1. General Appearance and Weight Loss

**Figure 1:**
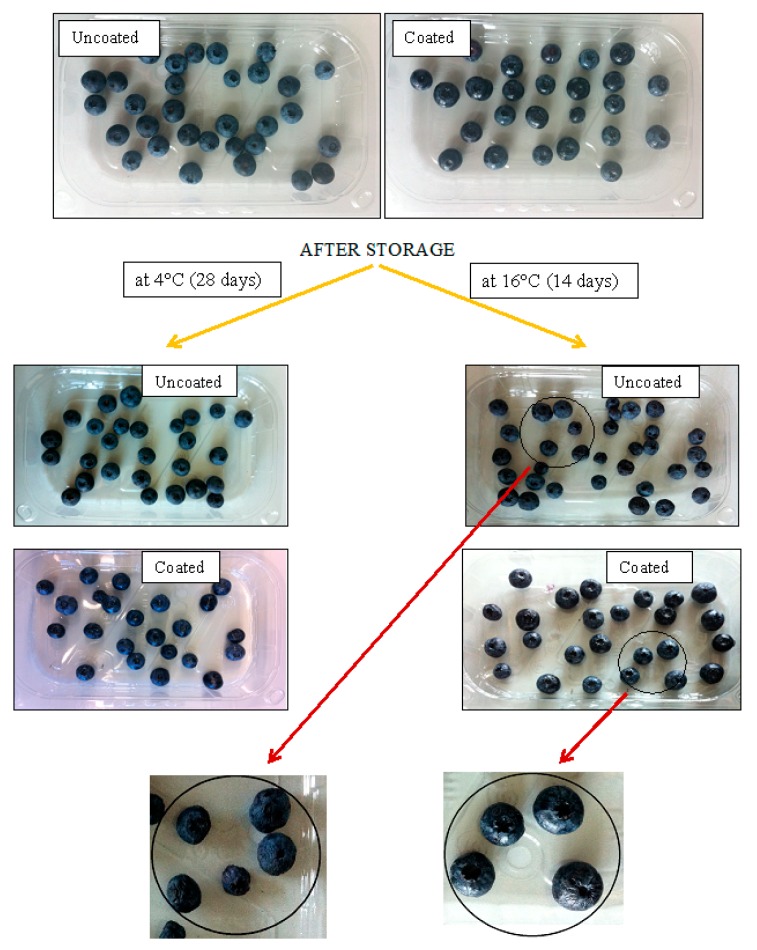
General appearance of blueberry samples before and after storage.

**Figure 2:**
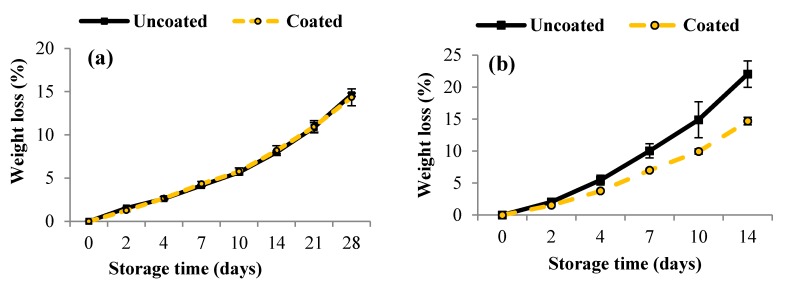
Weight loss of uncoated (C) and coated (P) blueberries storage at 4 °C for 28 days (**a**); and 16 °C for 14 days (**b**). Error bars represent the standard deviation. Values are means of three (*n* = 3) replications.

General appearance of uncoated and coated blueberries was visually evaluated before and after storage (Figure 1). In evaluating the general appearance of blueberries on day 0, we found that the surface of the coated fruits was bright and shiny. Pullulan coating was well adhered to the fruit during storage. During storage, unfavorable changes occurred in the general appearance of blueberries, mainly manifested by increased softening and wrinkling of the skin. These changes were apparent especially on uncoated blueberries. Pullulan-coated blueberries, however, were more protected against excessive fruit softening, thus preserving freshness and attractiveness for a long time. The intensity of progressive changes in the appearance of blueberries was influenced by the temperature of their storage. Blueberries stored at 16 °C had a more wrinkled skin compared to blueberries stored at 4 °C. Pullulan coating protected the fruit from loss of firmness and dehydration, especially when stored at higher temperatures, resulting in reduced weight loss. The effect of pullulan coating on changes in weight of blueberry is shown in Figure 2. As shown by the figure, pullulan coating had no statistically significant effect (*p* > 0.05) on changes in fruit weight of blueberries stored at 4 °C. After 28 days of storage at 4 °C, we found that the loss in fruit weight was up to 15% in uncoated and coated blueberries. In the case of blueberries stored at 16 °C, we found that the coated blueberries showed significantly lower (*p* < 0.05) weight loss than that of the uncoated blueberries. After 28 days of storage at 16 °C, fruit weight losses were found to be 14% and 22% for coated and uncoated samples, respectively. The lower pullulan effect in reducing the loss of blueberries at 4 °C compared to commercial temperature (16 °C) was probably due to higher relative humidity at 4 °C (RH > 95%) as opposed to 16 °C (RH 60–70%). 

Edible coatings are barriers that protect the fruits from excessive transpiration. Polysaccharide coatings exhibit a high barrier to oxygen, significantly limiting respiration of the fruit. According to the literature, one of the causes of increased fruit softening is the degradation of cell wall components such as the degradation of pectin by the pectinolytic enzymes. Slowing down the rate of respiration can limit the activity of this enzyme and delay the softening of fruits [28,29]. According to Chlebowska-Śmigiel et al. [30], pullulan coating on apples reduced the loss in fruit weight during storage. Wu and Chen [21] showed that pullulan coatings on apple slices significantly reduced weight loss during storage at 5 °C for 10 days. In addition, Eroglu et al. [28] studied the effect of different concentrations of pullulan coating on changes in the weight of strawberries. After 12 days of storage, the uncoated fruit weight of strawberries was found to be 19.65%, whereas the 8% and 10% pullulan coating resulted in reduced weight loss respectively to 16.58% and 14.91%. This difference in loss of weight is probably due to the thickness of pullulan coating. Thickness of pullulan coating increases as the concentration of pullulan is increased, thereby it protects the fruit from the loss of moisture from fruit [28]. The efficacy of pullulan coating in the storage of strawberry fruit has also been confirmed by Treviño-Garza et al. [31]. According to them, the use of pullulan coatings showed beneficial effects on the reduction of fruit weight loss.

### 2.2. Dry Matter (DM), Soluble Solid Content (SSC), and Reducing Sugar (Fructose and Glucose)

During storage, significant changes in chemical composition occur in the fruit due to the physiological processes (respiration, transpiration, or maturation). In this study, we investigated the effect of pullulan coating on changes in the basic parameters of blueberry affecting its quality. Changes in DM and SSC of coated and uncoated fruits during storage at 4 °C and 16 °C are shown in Table 1 and Table 2. The use of pullulan coating as a protective layer was found to be statistically significant (*p* < 0.05) in case of DM content of the fruit. After 28 days of storage at 4 °C, DM content of uncoated fruit increased by 20%, whereas DM content of coated fruits increased by 16% (Table 1). Similar changes in DM content of blueberries stored at 16 °C were observed in this study. After 2 weeks of storage, an increase in the DM content of uncoated and coated fruit was found to be 21% and 15%, respectively (Table 2). SSC is related to the degree of maturity of the fruit; fruit maturity accelerates metabolic reactions that increase the sugar content and sweetness of the fruit throughout storage [31]. As shown in Table 1 and Table 2, SSC of uncoated fruits stored at 4 °C was not significantly higher than that of coated fruit by the end of storage period (14.70 °Brix versus 14.80 °Brix, respectively; *p* > 0.05). Similar difference between uncoated and coated fruits with respect to SSC was observed in fruits stored at 16 °C. Compared to coated fruits, uncoated fruits showed higher SSC after storage, which is associated with metabolic processes such as maturation and overripening. The coated fruits had statistically significant (*p* < 0.05) lower SSC only in the first week of storage. Previous studies have reported that pullulan coating modified the atmosphere around the coated fruit, which in turn reduced the rate of respiration and thereby slowed the rate of metabolism [19,31,32].

**Figure 3:**
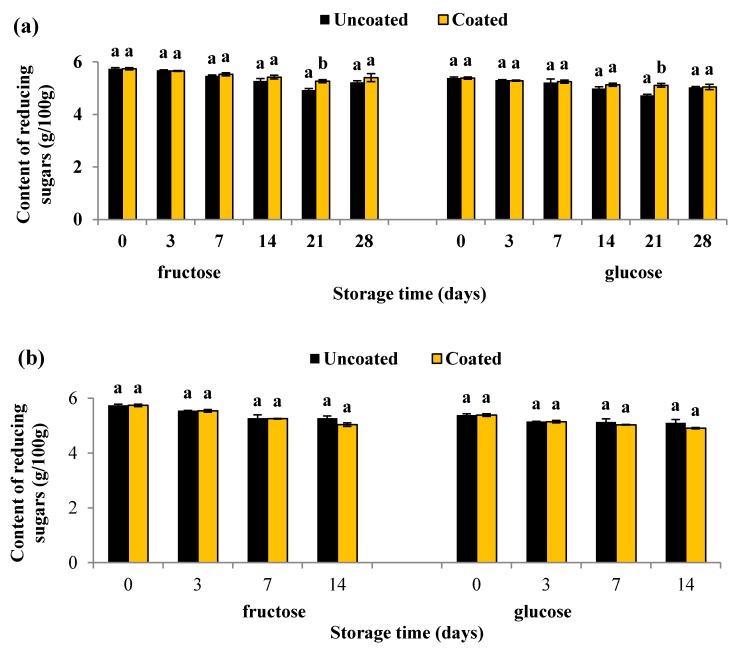
Reducing of sugar (fructose and glucose) content in uncoated and coated blueberries during storage at 4 °C for 28 days (**a**); and at 16 °C for 14 days (**b**). Error bars represent the standard deviation. Values are means of three (*n* = 3) replications. Means with different letters amongst different treatments for the same time are significantly different according to Tukey’s test (*p* < 0.05).

In this study, changes in the fruits’ SSC were correlated with the content of reducing sugars in blueberry fruits. Figure 3 shows changes in fructose and glucose content in uncoated and coated blueberry fruits during storage. Regardless of the storage temperature, there was a decrease in the content of reducing sugars. In the first two weeks of storage of blueberries at 4 °C, there were no statistically significant (*p* > 0.05) differences in the content of reducing sugars between coated and uncoated fruit. However, in the third week of storage, pullulan coating significantly reduced (*p* < 0.05) the content of reducing sugars. In the final fourth week of storage, there were no statistically significant (*p* > 0.05) differences in the content of reducing sugars between coated and uncoated fruits. Similarly, pullulan coating did not have a statistically significant (*p* > 0.05) effect on the change in reducing sugars in fruits stored at 16 °C.

According to this results, we can conclude that pullulan coating reduced the loss of reducing sugars (fructose and glucose) at a minor level in blueberries stored at 4 °C. The polar nature of polysaccharides affects their barrier properties. Polysaccharide coatings exhibit a high barrier toward gases such as oxygen and carbon dioxide. This high barrier enables the formation of pullulan coatings that reduce the rate of respiration of fruits and vegetables. During the respiration process in fruits, oxidation of carbohydrates occurs, accompanied by the release of carbon dioxide, water, and heat energy. Consequently, changes in carbohydrate content and total weight of fruit occur [19,33].

### 2.3. Content of L-Ascorbic Acid

**Figure 4:**
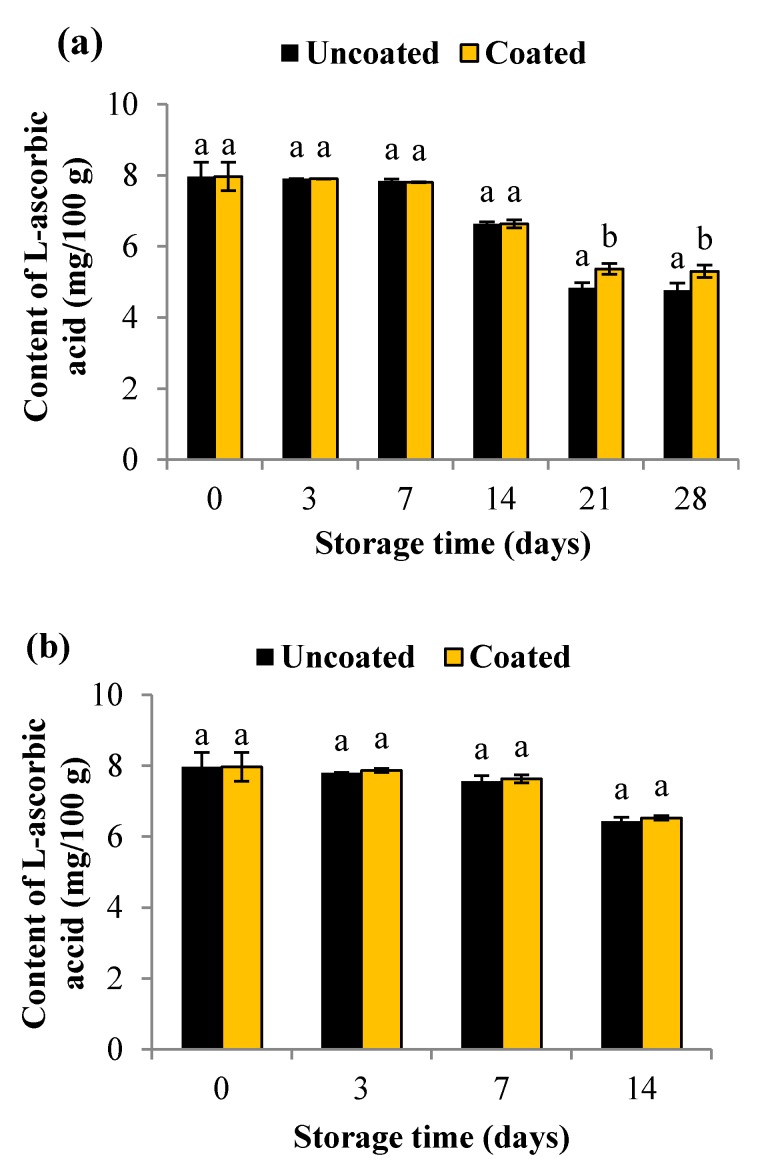
Content of vitamin C in uncoated and coated blueberries during storage at 4 °C for 28 days (**a**); and at 16 °C for 14 days (**b**). Error bars represent the standard deviation. Values are means of three (*n* 3) replications. Means with different letters amongst different treatments for the same time are significantly different according to Tukey’s test (*p* < 0.05).

The initial content of L-ascorbic acid in fresh blueberries was found to be 8 mg/100 g (Figure 4). During storage, regardless of the temperature, the content of L-ascorbic acid in fruit significantly decreased (*p* < 0.05). However, increased retention of L-ascorbic acid was observed in coated fruits. L-ascorbic acid content in uncoated fruits decreased by 40% after 28 days of storage at 4 °C, whereas at similar conditions, L-ascorbic acid content in coated fruits decreased by 33.5%. After 14 days of storage at 16 °C, the L-ascorbic acid content was found to be 19.3% and 18.06% in uncoated and coated fruits, respectively. These findings suggest that the edible coating used in this study helped in retaining the content of L-ascorbic acid in blueberries. Similar results were obtained by Treviño-Garza et al. [31] in strawberries coated with pectin-, pullulan-, and chitosan-based coatings. According to them, the delay in the reduction of L-ascorbic acid content can be attributed to reduced diffusion of oxygen, decreased rate of respiration, and consequently reduced oxidation.

### 2.4. Total Phenolic Content and Phytochemical Profiles

The effect of pullulan coating on the total content of polyphenols and individual phenolic acids in blueberry fruits is presented in Table 3 and Table 4. During storage, regardless of the temperature, there was a gradual increase in total content of polyphenols in blueberry fruit. The total content of polyphenols in uncoated and coated blueberries stored at 4 °C were found to be increased by 9.66% and 11.70%, respectively, whereas total polyphenols in uncoated and coated blueberries stored at 16 °C were found to be increased by 21.62% and 20.75%, respectively. Pullulan coating did not show any statistically significant effect on total polyphenol content in blueberry fruit (*p* > 0.05).

Among the phenolic acids present in blueberries, we found that chlorogenic acid was the dominant one (its initial content was 84.40 mg/100 g). The initial content of other phenolic acids, that is, caffeic and *p*-coumaric acid was found to be 16.20 mg/100 g and 10.47 mg/100 g, respectively. During storage, the content of phenolic acids was found to be increased. The greatest changes in the content of phenolic compounds have been reported with chlorogenic acid. After 28 days of storage of blueberries at 4 °C, the chlorogenic acid content in uncoated and coated blueberries was found to be 104.68 mg/100 g (24% more when compared to initial content) and 103.37 mg/100 g (22% more when compared to initial content), respectively. In turn, the increase in chlorogenic acid content in uncoated and coated fruits during the two weeks of storage at 16 °C was found to be 19.5% and 18.5%, respectively. Changes in the content of the remaining two identified phenolic acids (caffeic and *p*-coumaric acid) were within the range of 7–14% over the entire storage period. Nevertheless, we found a higher content of both phenolic acids (as well as chlorogenic acid) in uncoated blueberry than that of coated blueberries.

### 2.5. Anthocyanin Content

The highbush blueberry fruits are characterized by a diverse composition of anthocyanins. Different types of anthocyanins—including malividin, delphinidin, petunidin, cyanidin, and peonidin—were identified (Table 5 and Table 6). The glycosidic moiety of anthocyanins was formed by three monosaccharides: galactose, glucose, and arabinose. Blueberry fruits contained malvidin derivatives such as malvidin-3-galactoside and malvidin-3-arabinoside (over 18 mg/100 g). In addition, they also contained a high content of malvidin-3-glucoside, delphinidin-3-arabinoside, and delphinidin-3-galactoside, the average content of which ranged from 7.40 to 11.20 mg/100 g. According to the results, petunidin, cyanidin, peonidin and their derivatives are the minor components of all identified anthocyanins. Irrespective of the storage temperature of blueberries, a gradual increase in the content of individual anthocyanins was observed. Nevertheless, a slightly higher content of these pigments was found in uncoated fruit than in coated fruit.

Phenolic compounds present in blueberry fruit have antioxidant properties and are largely responsible for the health benefits of this fruit. They are present in the skin of the fruit, and their content in the fruit depends on the physiological state and the storage conditions [6,34,35,36]. In this study, increased contents of individual phenolic fractions—that is, phenolic acids and anthocyanins—were found in uncoated blueberries compared to pullulan-coated blueberries, which might be due to the increased transpiration and drying of the fruit than that of the coated fruit.

Phenolic compounds, found in the highest concentrations in the skin of the fruit, are strongly exposed to environmental factors, thereby causing faster degradation of these compounds than that of other compounds found in the skin. Pullulan coating creates an additional barrier around the skin of the coated fruit, thereby slowing down the intensity of the transpiration processes and reducing the degradation of phenolic compounds in the skin of blueberry. Kalt et al. [37] suggest that accumulation of phenolic acids and anthocyanins is sustained in overripened berries and in fruits after storage, because the loss of firmness of the outer layer enables the water to evaporate faster.

### 2.6. Decay Rate

**Figure 5:**
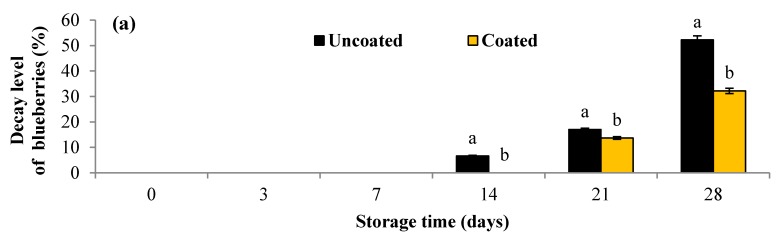

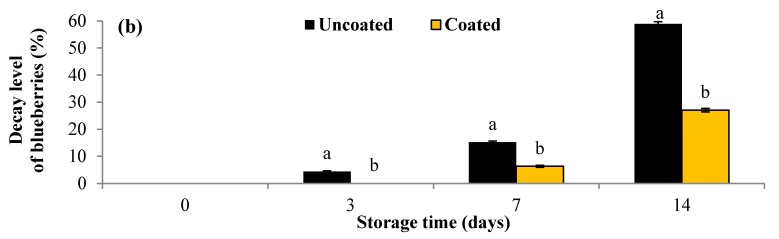
Evolution of the decay level (%) in pullulan coated blueberries during storage at 4 °C for 28 days (**a**); and at 16 °C for 14 days (**b**). Error bars represent the standard deviation. Values are means of three (*n* 3) replications. Means with different letters amongst different treatments for the same time are significantly different according to Tukey’s test (*p* < 0.05).

Effect of pullulan coating on the rate of decay in blueberries during 28 days of storage at 4 °C is presented in Figure 5. In general, the percentage of blueberries showing the signs of decay was lower in the coated fruits than that of uncoated fruits during the storage period. Until day 7 of storage, uncoated as well coated fruits showed good signs of hydration and color, and signs of decay in samples were not observed. Decay and contamination of fruit first appeared on uncoated samples on day 14. At day 21, the percentage of decay of coated blueberries was 13.70%, which was still significantly less (*p* < 0.05) than that of uncoated samples 17.04%. After 28 days of storage, the percentages of fruits showing signs of decay in uncoated and coated fruits were 52.22% and 32.22%, respectively. Similar results were observed at a storage temperature of 16 °C (Figure 5). The percentage of fruits showing signs of decay in pullulan-coated fruits was significantly less (*p* < 0.05) than that of uncoated fruits throughout the storage period. At day 14, the percentage of blueberries coated with pullulan was 27.04% and was still significantly less (*p* < 0.05) than that of uncoated fruits (58.89%).

Thus, we can conclude that the application of pullulan coating was efficient in preserving the quality of blueberries; it protected the fruits from decay when compared to the uncoated samples. Moreover, uncoated blueberries were found to be dehydrated and were largely contaminated by the growth of mold at the end of storage period, whereas pullulan-coated blueberries showed good hydration and color, which implies a promising attribute for consumption (Figure 1).

Blueberries are susceptible to mechanical damage, which in turn contributes to their rapid deterioration. Development of microflora, in particular mold and bacteria, is responsible for spoilage and the reduction of persistence in fruits [12,38]. Pullulan coating significantly reduced the changes related to delayed damage by mold and helped to control microbial spoilage.

Polysaccharide coatings are hydrophilic polymers that are characterized by limited gas permeability, mainly oxygen and carbon dioxide [39,40]. Low oxygen permeability is responsible for the inhibition of growth of mold [41,42,43]. Thus, it can be concluded that the mechanism of action of such a coating is to mechanically cut off access to atmospheric oxygen [22]. This limitation to oxygen adversely affects the development of microorganisms with high oxygen requirements, such as molds. This has been confirmed by studies performed by Eroglu et al. [28] where strawberries were coated with pullulan (at 8% and 10% concentration). According to them, the coating on the strawberries delayed the growth of mold on the surface of the fruit on an average by three to five days compared to the uncoated fruit. Kraśniewska et al. [27] coated the sprouts of Brussels with pullulan and found a significant reduction in the number of *Aspergillus niger* in these samples. The positive influence of pullulan coatings on fruit and vegetable storage quality has also been confirmed by other studies [23,44]. For example, pullulan coating of paprika and apples significantly reduced the rotting process of fruit contaminated with *A. niger* spores, stored at ambient temperature. A similar trend was observed by Synowiec et al. [45] who successfully used pullulan coating to protect the surface of apples from the development of *Rhizopus arrhizus*, thereby significantly inhibiting the development of mold responsible for apple rot.

## 3. Materials and Methods

### 3.1. Materials

Pullulan was obtained from Carbosynth (Compton, UK). Highbush blueberry fruits (*Vaccinium corymbosum* L. cv. Bluecrop) were harvested during September 2015 from local growers near Warsaw in Poland (51°99′ N, 21°00′ E). After harvest, fruits were stored at 4 °C for 12 h until the coating was applied. Blueberries of uniform size, shape, color, and without any signs of mechanical damage or fungal decay were selected.

### 3.2. Preparation and Application of Pullulan Coating

Pullulan coating solution was prepared by dissolving pure pullulan 10% (w/v) and glycerol 1% (w/v) (POCH S.A., Gliwice, Poland) in distilled water. The components were mixed with a magnetic stirrer (500 rpm) for 20 min (ES-21, WIGO, Pruszków, Poland). Glycerol was added as a plasticizer to improve the mechanical properties of the coating material.

Fruits were randomly distributed into one of the two groups: those to be coated with pullulan and those that remain uncoated. Blueberries were dipped in pullulan coating solution for 3 min and dried at room temperature (20 °C) in a laminar flow cabinet (ESCO, Singapore) for 1 h at a relative humidity of 55–60%. Uncoated samples of blueberries (control) were dipped in deionized water and also dried. Then uncoated and coated samples of fruits were placed inside commercial PET (polyethylene terephthalate), vented clamshell boxes. All samples were stored in cold stores and chamber respectively at 4 ± 1 °C for 28 days and at 16 ± 2 °C for 14 days.

### 3.3. Physicochemical Analysis

#### 3.3.1. Weight Loss

Weight loss was determined by weighing the uncoated and coated samples every other day throughout the storage period. Results were expressed as the percentage of weight lost by using the formula

$$\text{Weight loss}\;(\%)\;=\;\frac{w_{i}-w_{f}}{w_{i}}\times 100$$

where, *w_i_* is the initial weight of fruits and *w_f_* is the weight of fruits during storage.

#### 3.3.2. DM and SSC

DM was determined by drying the ground fruits in a vacuum oven at 70 °C until a constant weight was obtained. The total SSC (°Brix) in samples was measured at 20 °C by digital refractometer (Model 30PX, Mettler-Toledo AG, Columbus, OH, USA).

#### 3.3.3. Reducing Sugars

Determination of sugars in fruit samples was performed using the Agblevor et al. [46] method with some modification. The sample (10 g) was homogenized with 80 mL of distilled water using Ultra-Turrax homogenizer (IKA, Wilmington, DE, USA). The extract was collected into a volumetric flask of 100 mL and then 2 mL of 2% calcium hydroxide was added to neutralize the pH. The level of this solution in the flask was made up to the mark with the use of distilled water. The sample was centrifuged at 10,000 rpm for 10 min and filtered using a polyamide (PA) syringe filters with a pore size of 0.45 µm. Fructose and glucose content were determined by high performance liquid chromatography (HPLC) using a Shimadzu apparatus with a refractive index detector and carbohydrate analysis column (300 mm × 3.9 mm, Waters, Milford, MA, USA). The eluent was the solution of acetonitrile and water (800:200, v/v), at a flow rate of 1.5 mL/min at 25 °C. A quantitative analysis of glucose and fructose was performed based on calibration curves for standard solutions.

#### 3.3.4. Content of L-Ascorbic Acid 

L-ascorbic acid content was determined by HPLC using a Shimadzu apparatus (LC-10AT, Shimadzu, Kyoto, Japan) consisting of a quaternary solvent pumping system (FCV-10ALVP, Shimadzu, Kyoto, Japan), degasser model DG-4400 (Shimadzu, Kyoto, Japan), UV–Vis detector (SPD-10AVP, Shimadzu, Kyoto, Japan), column oven (CTO-10ASVP, Shimadzu, Kyoto, Japan), autosampler (SIL-20AHT, Shimadzu, Kyoto, Japan), and an LC solution data collection system (Shimadzu, Kyoto, Japan). A ZIC^®^-HILIC column (4.6 mm × 150 mm, 5 µm, SeQuant, Umeå, Sweden) was used to measure the concentration of L-ascorbic acid in fruit according to the method of Drivelos et al. [47] with some modifications. Homogenized sample (2–5 g) was extracted in 40 mL of 10 mM oxalic acid solution. The extract was centrifuged at 3200 rpm for 10 min at 4 °C and the supernatant was added to the volumetric flask and made up to the volume using distilled water. The sample was diluted with acetonitrile: water with 66.7 mM ammonium acetate (850:150 v/v) solution by mixing one part of the sample with one part of diluent solution. Sample was filtered through PTFE 0.45 µm filter prior to analysis. The chromatographic analyses were performed using a solution of acetonitrile:water (850:150, v/v) with 66.7 mM ammonium acetate as the mobile phase. Flow rate was adjusted to 0.5 mL/min, and the absorbance was monitored at 240 nm. A calibration curve was constructed using freshly prepared L-ascorbic acid solution (in the range of 1–50 µg).

#### 3.3.5. Total Phenolic Content

Total phenolic content was determined by the Folin–Ciocalteu method [48] using chlorogenic acid as the standard. Fruits (10 g) were homogenized with 40 mL of solution containing acetone:methanol:water (350:350:300) using Ultra-Turrax homogenizer (IKA, Wilmington, DE, USA). The sample was centrifuged at 5000 rpm for 10 min, and the supernatant was collected into a volumetric flask (200 mL). The extraction was repeated twice and then the solution was made up to the volume of 200 mL using distilled water. Next, 100 µL of this extract was mixed with 200 µL of Folin–Ciocalteu reagent and incubated for 3 min. Then, 2 mL of distilled water and 1 mL of 15% sodium carbonate were added. The mixture was incubated at room temperature, and the absorbance of the sample was measured at 765 nm after 60 min incubation. A calibration curve was performed with chlorogenic acid, and the results were expressed as milligrams of chlorogenic acid equivalents per 100 g of fruit.

#### 3.3.6. Quantification of Anthocyanins and Phenolic Acids

The content of anthocyanins and phenolic acids was determined by HPLC using Shimadzu apparatus and diode array detector (SPD-M20A). Briefly, 10 g of homogenized fruit was extracted thrice with methanol/water/hydrochloric acid solution (700/300/1, v/v/v). The sample was completely evaporated of the methanol and brought to a volume of 50 mL using an aqueous phosphoric acid solution (1.0 g/L). The sample was centrifuged at 5000 rpm for 10 min, and the preconditioned Sep-Pak C_18_ cartridge was used for purification of the extract. Anthocyanins and phenolic acids were adsorbed into the cartridge while organic acids, sugars, and other compounds were removed. Samples were filtered through PTFE 0.45 µm filter before HPLC analyses. Separation of anthocyanins and phenolic acids was performed on a Luna C18(2) RP (5 µm) 250 × 4.6 column (Phenomenex, Torrance, CA, USA). These compounds were eluted with a gradient of phase A (water:formic acid, 900:100, v/v) and phase B (acetonitrile:formic acid, 900:100, v/v) starting with 5% phase B, then 8% phase B for 8 min, 11% phase B for 10 min, 14% phase B for 3 min, 22% phase B for 5 min, 32% phase B for 4 min, and 5% phase B for 4 min used at the flow rate of 1 mL/min. Peak areas were monitored at 520 nm (anthocyanins) and 320 nm (phenolic acids). Monomeric anthocyanins and phenolic acids were identified by comparing retention time, UV–Vis spectra, and elution order against authentic standards or against previously reported data. Monomeric anthocyanins were quantified by comparing their peak areas in the chromatograms at 520 nm with the peak area of malvidin-3-glucoside. A quantitative analysis of phenolic acid was performed based on calibration curves for standard solutions.

### 3.4. Microbiological Characteristics of Fruit Decay

Uncoated and coated blueberries were visually examined during storage period. Fruits which showed surface mycelial development or bacterial lesions were considered as decayed. Fruit decay was expressed as the percentage of fruit showing decay symptoms.

### 3.5. Statistical Analysis

The results were reported as mean with standard deviations. One-way analysis of variance (ANOVA) was used to process the data. The means were compared using Tukey’s range tests at *p* < 0.05. Calculations were performed with Statistica 12 software (StatSoft Inc., Statistica 12, Tulsa, OK, USA).

## 4. Conclusions

During storage of fruits there are a number of physiological processes related to their maturation and overripening which may adversely affect the sensory properties and attractiveness of the raw material, thereby reducing their quality and commercial value. The primary causes of reduced commercial value of fresh plant materials are quantitative losses due to respiration and wilting due to water loss, nutrient depletion in the respiration process, and microbial spoilage. Based on the research, we can conclude that the coated fruits of blueberries were more protected against excessive sagging and crinkling of the skin. Pullulan coating was also found to reduce the drying and wilting of the raw material, especially at higher temperatures, thereby preserving its freshness and attractiveness for an extended period. Coating of blueberry fruit with pullulan resulted in a decrease in weight loss and a reduction in the change in the content of extract and reducing sugars, which was probably the result of a decrease in the rate of respiration and transpiration. In addition, a higher retention of L-ascorbic acid was observed in the coated fruits. In turn, changes in the content of polyphenols and anthocyanins—the compounds occurring in the highest quantities in the skin of the fruit—were correlated with the loss of weight in the examined raw material. With the greater weight loss in uncoated fruit, a higher phenolic content was observed. In addition, the coating of blueberries significantly reduced the changes related to the rotting of blueberries.

## Figures and Tables

**Table 1:** Dry matter (%), soluble solids content (SSC) (°Brix) in uncoated and coated blueberries fruit. Fruit were stored at 4 °C for up to 28 days.

Quality Parameters	Days	Uncoated	Coated
Dry matter (%)	0	16.58 ± 0.03 a	16.58 ± 0.03 a
	7	17.49 ± 0.06 a	16.68 ± 0.06 b
	14	18.51 ± 0.06 a	17.70 ± 0.06 b
	21	18.92 ± 0.06 a	18.78 ± 0.02 b
	28	19.88 ± 0.08 a	19.29 ± 0.06 b
SSC (°Brix)	0	13.20 ± 0.10 a	13.20 ± 0.10 a
	7	13.57 ± 0.06 a	13.23 ± 0.15 b
	14	14.50 ± 0.10 a	14.07 ± 0.12 b
	21	14.70 ± 0.10 a	14.77 ± 0.06 a
	28	14.70 ± 0.10 a	14.80 ± 0.10 a

Each value is the mean for three (*n* 3) replicates. Means with the different letters within the same row are significantly different according to Tukey’s test (*p* < 0.05).

**Table 2:** Dry mass (%), soluble solids content (SSC) (°Brix) in uncoated and coated blueberries fruit. Fruit were stored at 16 °C for up to 14 days.

Quality Parameters	Days	Uncoated	Coated
Dry matter (%)	0	16.58 ± 0.03 a	16.58 ± 0.03 a
	3	16.71 ± 0.03 a	16.76 ± 0.15 a
	7	17.64 ± 0.05 a	16.93 ± 0.03 b
	14	20.04 ± 0.09 a	19.04 ± 0.05 b
SSC (°Brix)	0	13.20 ± 0.10 a	13.20 ± 0.10 a
	3	13.27 ± 0.06 a	13.33 ± 0.12 b
	7	13.80 ± 0.10 a	13.47 ± 0.06 b
	14	14.80 ± 0.10 a	14.80 ± 0.10 a

Each value is the mean for three (*n* 3) replicates. Means with the different letters within the same row are significantly different according to Tukey’s test (*p* < 0.05).

**Table 3:** Contents of total phenolics and phenolic acids in uncoated and coated blueberries fruit. Fruit were stored at 4 °C for up to 28 days.

Quality Parameters	Days	Uncoated	Coated
TPC (mg CAE/100 g)	0	268.47 ± 6.16 a	268.47 ± 6.16 a
	28	294.13 ± 2.11 a	300.07 ± 6.89 a
Phytochemicals (mg/100 g)			
Phenolic acids			
Chlorogenic acid	0	84.40 ± 2.30 a	84.40 ± 2.30 a
	7	89.57 ± 0.48 a	87.62 ± 0.42 b
	14	97.55 ± 0.28 a	97.27 ± 0.55 a
	21	99.43 ± 0.86 a	100.23 ± 0.43 a
	28	104.68 ± 1.04 a	103.37 ± 0.38 a
Caffeic acid	0	16.20 ± 0.62 a	16.20 ± 0.62 a
	7	16.63 ± 0.07 a	15.82 ± 0.06 b
	14	17.17 ± 0.10 a	16.35 ± 0.11 a
	21	17.24 ± 0.13 a	17.42 ± 0.20 a
	28	17.92 ± 0.16 a	17.78 ± 0.26 a
*p*-Coumaric	0	10.47 ± 0.70 a	10.47 ± 0.70 a
	7	10.71 ± 0.05 a	10.19 ± 0.03 b
	14	11.02 ± 0.08 a	10.49 ± 0.08 b
	21	11.09 ± 0.03 a	10.87 ± 0.05 b
	28	11.52 ± 0.04 a	11.04 ± 0.09 b

Each value is the mean for three (*n* 3) replicates. Means with different letters within the same row are significantly different according to Tukey’s test (*p* < 0.05).

**Table 4:** Contents of total phenolics and phenolic acids in uncoated and coated blueberries fruit. Fruit were stored at 16 °C for up to 14 days.

Quality Parameters	Days	Uncoated	Coated
TPC (mg CAE/100 g)	0	268.47 ± 6.16 a	268.47 ± 6.16 a
	14	326.50 ± 0.87 a	324.17 ± 2.98 a
Phytochemicals (mg/100 g)			
Phenolic acids			
Chlorogenic acid	0	84.40 ± 2.30 a	84.40 ± 2.30 a
	3	87.06 ± 0.19 a	87.61 ± 0.95 a
	7	91.02 ± 0.60 a	89.85 ± 0.14 b
	14	104.01 ± 0.78 a	103.80 ± 0.23 a
Caffeic acid	0	16.20 ± 0.62 a	16.20 ± 0.62 a
	3	15.99 ± 0.03 a	15.98 ± 0.13 a
	7	16.65 ± 0.09 a	15.90 ± 0.07 b
	14	18.34 ± 0.16 a	17.05 ± 0.13 b
*p*-Coumaric	0	10.47 ± 0.70 a	10.47 ± 0.70 a
	3	10.30 ± 0.06 a	10.30 ± 0.08 a
	7	10.71 ± 0.07 a	10.22 ± 0.05 b
	14	11.75 ± 0.11 a	11.04 ± 0.10 a

Each value is the mean for three (*n* 3) replicates. Means with different letters within the same row are significantly different according to Tukey’s test (*p* < 0.05).

**Table 5:** Contents of anthocyanins in uncoated and coated blueberries fruit. Fruit were stored at 4 °C for up to 28 days.

Storage Time	Anthocyanins [mg/100 g]														
	Mv-3-gal	Mv-3-glu	Mv-3-ara	Dp-3-gal	Dp-3-glu	Dp-3-ara	Pt-3-gal	Pt-3-glu	Pt-3-ara	Cy-3-gal	Cy-3-glu	Cy-3-ara	Pn-3-gal	Pn-3-glu	Acyl
Uncoated															
0	18.47 ± 0.55	11.20 ± 0.61	18.47 ± 0.81	7.40 ± 0.20	4.07 ± 0.06	8.13 ± 0.21	5.63 ± 0.23	3.60 ± 0.17	4.30 ± 0.17	0.63 ± 0.06	0.50 ± 0.01	0.60 ± 0.01	0.30 ± 0.01	0.63 ± 0.06	12.93 ± 0.31
14	21.33 ± 0.21	13.00 ± 0.17	21.30 ± 0.70	8.87 ± 0.06	4.87 ± 0.12	9.80 ± 0.17	6.53 ± 0.15	4.07 ± 0.15	4.87 ± 0.15	0.70 ± 0.01	0.53 ± 0.06	0.70 ± 0.01	0.40 ± 0.06	0.70 ± 0.01	15.63 ± 0.42
28	23.07 ± 0.32	14.07 ± 0.25	23.50 ± 0.26	9.77 ± 0.15	5.20 ± 0.10	10.30 ± 0.20	7.13 ± 0.12	4.50 ± 0.10	5.37 ± 0.06	0.80 ± 0.01	0.60 ± 0.01	0.73 ± 0.06	0.40 ± 0.01	0.80 ± 0.01	16.53 ± 0.31
Coated															
0	18.47 ± 0.55	11.20 ± 0.61	18.47 ± 0.81	7.40 ± 0.20	4.07 ± 0.06	8.13 ± 0.21	5.63 ± 0.23	3.60 ± 0.17	4.30 ± 0.17	0.63 ± 0.06	0.50 ± 0.01	0.60 ± 0.01	0.30 ± 0.01	0.63 ± 0.06	12.93 ± 0.31
14	20.57 ± 0.55	12.70 ± 0.06	21.00 ± 0.61	8.37 ± 0.15	4.67 ± 0.15	9.27 ± 0.25	6.37 ± 0.23	4.00 ± 0.17	4.70 ± 0.17	0.70 ± 0.01	0.50 ± 0.01	0.67 ± 0.06	0.37 ± 0.06	0.70 ± 0.01	14.80 ± 0.40
28	22.70 ± 0.30	13.67 ± 0.21	21.80 ± 0.26	9.27 ± 0.06	5.20 ± 0.01	10.43 ± 0.12	6.73 ± 0.06	4.17 ± 0.06	4.97 ± 0.12	0.80 ± 0.01	0.60 ± 0.01	0.70 ± 0.01	0.40 ± 0.01	0.70 ± 0.01	16.57 ± 0.12

Each value is the mean for three (*n* 3) replicates.

**Table 6:** Contents of anthocyanins in uncoated and coated blueberries fruit. Fruit were stored at 16 °C for up to 14 days.

Storage Time	Anthocyanins [mg/100 g]														
	Mv-3-gal	Mv-3-glu	Mv-3-ara	Dp-3-gal	Dp-3-glu	Dp-3-ara	Pt-3-gal	Pt-3-glu	Pt-3-ara	Cy-3-gal	Cy-3-glu	Cy-3-ara	Pn-3-gal	Pn-3-glu	Acyl
Uncoated															
0	18.47 ± 0.55	11.20 ± 0.61	18.47 ± 0.81	7.40 ± 0.20	4.07 ± 0.06	8.13 ± 0.21	5.63 ± 0.23	3.60 ± 0.17	4.30 ± 0.17	0.63 ± 0.06	0.50 ± 0.01	0.60 ± 0.01	0.30 ± 0.01	0.63 ± 0.06	12.93 ± 0.31
7	21.63 ± 0.38	13.37 ± 0.23	22.33 ± 0.55	8.93 ± 0.12	4.87 ± 0.06	9.80 ± 0.17	6.80 ± 0.17	4.27 ± 0.06	5.07 ± 0.06	0.77 ± 0.06	0.57 ± 0.06	0.70 ± 0.01	0.40 ± 0.01	0.77 ± 0.06	15.63 ± 0.29
14	23.77 ± 0.38	14.70 ± 0.10	24.17 ± 0.15	9.63 ± 0.21	5.23 ± 0.12	10.47 ± 0.23	7.37 ± 0.06	4.67 ± 0.06	5.57 ± 0.06	0.80 ± 0.01	0.60 ± 0.01	0.77 ± 0.06	0.40 ± 0.01	0.80 ± 0.01	16.70 ± 0.36
Coated															
0	18.47 ± 0.55	11.20 ± 0.61	18.47 ± 0.81	7.40 ± 0.20	4.07 ± 0.06	8.13 ± 0.21	5.63 ± 0.23	3.60 ± 0.17	4.30 ± 0.17	0.63 ± 0.06	0.50 ± 0.01	0.60 ± 0.01	0.30 ± 0.01	0.63 ± 0.06	12.93 ± 0.31
7	21.17 ± 0.38	12.87 ± 0.25	21.13 ± 0.42	8.63 ± 0.06	4.80 ± 0.10	9.53 ± 0.15	6.40 ± 0.10	4.00 ± 0.10	4.77 ± 0.15	0.70 ± 0.01	0.53 ± 0.06	0.70 ± 0.01	0.40 ± 0.01	0.70 ± 0.01	15.27 ± 0.31
14	22.90 ± 0.17	14.10 ± 0.40	23.57 ± 0.80	9.57 ± 0.21	5.13 ± 0.06	10.30 ± 0.01	7.17 ± 0.21	4.53 ± 0.15	5.37 ± 0.15	0.80 ± 0.01	0.60 ± 0.01	0.70 ± 0.01	0.40 ± 0.01	0.80 ± 0.01	16.47 ± 0.06

Each value is the mean for three (*n* 3) replicates.

## Abbreviations

The following abbreviations are used in this manuscript:

Mv-3-gal	malvidin 3-galactoside
Mv-3-glu	malvidin 3-glucoside
Mv-3-ara	malvidin 3-arabinoside
Dp-3-gal	delphinidin-3-galactoside
Dp-3-glu	delphinidin 3-glucoside
Dp-3-ara	delphinidin 3-arabinoside
Pt-3-gal	petunidin-3-galactoside
Pt-3-glu	petunidin 3-glucoside
Pt-3-ara	petunidin 3-arabinoside
Cy-3-gal	cyaniding-3-galactoside
Cy-3-glu	cyaniding-3-glucoside
Cy-3-ara	cyaniding-3-arabinoside
Pn-3-gal	Peonidine-3-galactoside
Pn-3-glu	Peonidine-3-glucoside
Acyl	acylated anthocyanins.
